# Cross-Cultural Adaptation and Validation of the Arabic Version of the Mini-BESTest among Community-Dwelling Older Adults in Saudi Arabia

**DOI:** 10.3390/healthcare10101903

**Published:** 2022-09-28

**Authors:** Bader A. Alqahtani, Ahmed S. Alhowimel, Mohammed M. Alshehri, Maha A. Alqahtani, Abdulaziz A. Almuhaysh, Ali O. Alshakarah, Aljawharah Alanazi, Aeshah H. Khoja, Aqeel M. Alenazi

**Affiliations:** 1 Department of Health and Rehabilitation Sciences, Prince Sattam Bin Abdulaziz University, Al-Kharj 11942, Saudi Arabia; 2 Physical Therapy Department, Jazan University, Jazan 82775, Saudi Arabia; 3 Research and innovation department, King Abdullah International Medical Research Center, Riyadh 13223, Saudi Arabia; 4 Physical Therapy Department, Ministry of Health Durma General Hospital, Riyadh 1472, Saudi Arabia; 5 Physical Therapy Department, Ministry of Health Afif General Hospital, Al-Kharj 11942, Saudi Arabia; 6 Physical Therapy Department, Ministry of Health Diriyah Hospital, Riyadh 14245, Saudi Arabia; 7 Physical Therapy Department, Saudi German Hospital, Riyadh 42317, Saudi Arabia

**Keywords:** Mini-BESTest, Arabic, balance

## Abstract

Backgrounds: The Mini-BESTest is a clinical assessment of balance impairment; however, the translation and psychometric properties in the Arabic-speaking population have not yet been investigated. The purpose of this study was to translate into Arabic and evaluate the psychometric properties of the Mini-BESTest in Saudi community-dwelling older adults. Methods: This is a cross-sectional transcultural adaptation and validation study. A total of 144 community-dwelling older adults were enrolled (mean age = 66.2 ± 6.2 years). The translation and cross-cultural adaptation of the Mini-BESTest from English to Arabic was performed using standardized guidelines. Test–retest reliability was examined using the intraclass correlation coefficient (ICC) with one week between test and retest. The internal consistency was assessed using Cronbach’s alpha. Construct validity of the Mini-BESTest was assessed using balance such as Berg Balance Scale (BBS) and Falls Efficacy Scale International (FES-I). Results: The Arabic version of the Mini-BESTest showed good internal consistency (Cronbach’s alpha = 0.93). The scale shows excellent test–retest reliability (ICC = 0.99, 95% CI, 0.98–0.99) and excellent inter-rater reliability (ICC = 0.93, 95% CI, 0.70–0.97), which is indicative of the measure’s stability and repeatability. Mini-BESTest total scores showed an excellent inter-rater agreement. There was a significant correlation between total score of the Mini-BESTest and BBS (*r* = 0.72; *p* < 0.001). Mini-BESTest had a moderate association with FES-I. Conclusion: The Arabic version of the Mini-BESTest is a reliable and valid test for assessing balance in older adults. More research is needed to confirm the test’s reliability and validity in a specific population, such as those with neurological problems.

## 1. Introduction

The global population of people aged ≥65 years was 703 million in 2019. This population of older people is expected to reach 1.5 billion in 2050. The global proportion of people aged ≥65 years has increased from 6% in 1990 to 9% in 2019, and this percentage is expected to reach 16% by 2050 [1]. This rapid growth of the aging population can be seen in many countries around the world including Arab countries. Life expectancy has also increased globally and regionally in countries such as Saudi Arabia, leading to a steady increase in the proportion of older adults [2]. In 2014, the average life expectancy was 74.8 years (approximately 72.8 years for men and 76.9 years for women). The World Health Organization reported that 4.3% of the population in the Saudi Arabia was between 55 and 64 years of age. According to the United Nations, the population of Saudi Arabians aged ≥65 years will continue to increase and will account for 18.4% of the country’s population in 2050. In light of this prediction, there is a growing interest in studying aging and age-related health outcomes among older Saudi adults [3]. 

Aging is associated with the deterioration or decline of the functions of different body systems, such as the musculoskeletal and cardiovascular systems. The balance function of the body naturally declines with age due to the loss of sensory and musculoskeletal functions and the inability to integrate information. Balance can also further deteriorate because of diseases that are common in the older population [4]. Roughly one-third of older adults who are over 65 years old fall annually. Falls are linked to higher rates of morbidity and mortality as well as contributing to medical care expenses. Among the many other risk factors that have been found, loss of balance is a significant modifiable risk factor for falls. Therefore, there is a need to establish easily administered tools to assess the balance clinically during regular checking up for older adults.

Balance disorders can be assessed manually through a commonly used tool called the Mini Balance Evaluation Systems Test (Mini-BESTest). The original BESTest was designed by Horak et al. in 2009 [5], and showed easy utilization with excellent reliability and validity [6]. The Mini-BESTest appears to be superior to other balance tests because of the illegibility of dynamic and static balance measures. The development of the Mini-BESTest was based on balance and mobility evaluation via identifying position control systems and poor functional balance. There are four categories of Mini-BESTest including reparatory adjustments, reactive posture control, sensory orientation, and dynamic gait, which have made it possible to identify the compromised system in various health conditions. Nevertheless, its long-term clinical benefit still requires further evaluation. Therefore, in 2010, Franchignoni et al. developed a shorter version, called the Mini-BESTest, using a Rasch analysis [7]. Since the Mini-BESTest is a newly developed balance test, it needs further evaluation for cross-cultural adaption in different populations.

Compared to other balance tests, the reliability, responsiveness, and content validity of the psychometric properties of the Mini-BESTest are excellent with regard to different diseases, such as stroke and Parkinson’s disease (PD). Additionally, the Mini-BESTest has no significant floor and ceiling effects in patients with different illnesses and disease severity, with excellent inter- and intra-rater reliability [8,9]. The normative values for the Mini-BESTest scores are age-dependent and have been shown to decrease with age [10]. In addition, the Mini-BESTest has a strong and highly significant correlation with other balance and gait measures for patients with PD and other neurological disorders and it is highly predictive of falls in patients with PD and stroke [11]. The Mini-BESTest and BESTest are also commonly used to assess fall risk in healthy older adults of different ages as well as those with the aforementioned illnesses [8]. Compared with other measurements such as the BBS, the Timed Up and Go (TUG) test, and gait speed, the Mini-BESTest presents a more rapid assessment of dynamic balance and control [10]. The psychometric approaches of the BBS, based on the item response theory, mainly include balance and mobility scores, but do not include other sub-optimal functional categories. In contrast, the Mini-BESTest scale has excellent psychometric properties, which can be clinically administered [7].

The original Mini-BESTest was written in English and has been translated into different languages, such as Greek [12], Spanish [13], German [14], Swedish [15], French [16], Italian [7], Persian [17], and Turkish [18]. However, there is currently no Arabic translation for the test, which may limit its use in the Arabic-speaking population. Therefore, this study aimed to translate and culturally adapt the Mini-BESTest into Arabic for its application in Saudi Arabia. We hypothesized that the Arabic version of the Mini-BESTest would show comparable results to those of the original version, with good inter-rater and test–retest reliability and validity.

## 2. Materials and Methods

### 2.1. Study Design and Participants

This cross-sectional study was conducted in Riyadh and the Madinah area. Older community-dwellers were recruited to participate in the study. An approved informed consent form was signed by all participants. The participants, mostly retirees, included Saudi Arabian citizens and Arab residents in Saudi Arabia at the time of the interview. Participants were eligible if they were Arabic speakers, aged 60 years or older, able to walk independently, and able to follow simple instructions. Participants with any acute medical conditions or a cognitive function impairment score of <24 out of 30 on the Mini-Mental State Examination were excluded. 

### 2.2. Transcultural Translation Process

**Figure 1:**
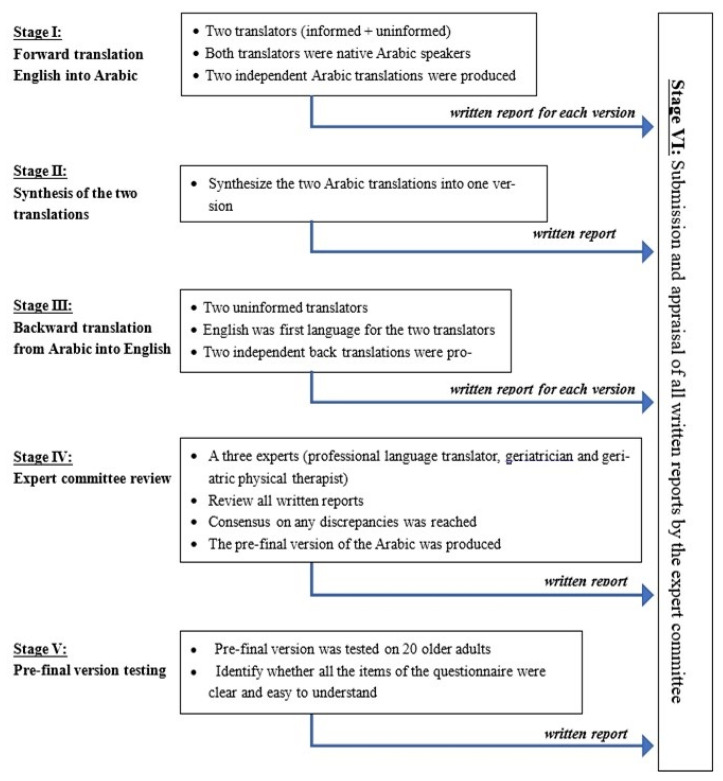
Flowchart of the cross-cultural adaptation process of the Arabic version of the Mini-BESTest.

Our translation of the Mini-BESTest included a five-step translation process that followed the international guidelines by Beaton [19]. In the first step, “forward translation”, the test was translated by two separate bilingual translators who were domestic, native Arabic speakers. In the second step, a synthesis of the two translations in the previous step was created by consensus. In the third step, “backward translation”, the synthesized version was translated back into English by two bilingual independent translators who were domestic, native Arabic speakers and blinded to the English version of the Mini-BESTest. In the fourth step, another cross-cultural validation of the backward translations of the Mini-BESTest was undertaken to generate a final Arabic version of the Mini-BESTest. Finally, the pre-final version the TFI scale was tested on a group of older adults (n = 20) to assess if all the test items were clear and easy to understand. The pre-final version was approved and ready to be used. Each participant understood the translated version of the test clearly and showed no difficulties in following instructions and understanding the items of the scale (Figure 1).

### 2.3. Outcome Measures

The Mini-BESTest is a 14-item test, designed to assess dynamic balance within 10–15 min, excluding the biomechanical and stability items. The 4 sections of this test included (1) anticipatory postural adjustment, (2) reactive postural control, (3) sensory orientation, and (4) dynamic gait balance. Two items, right and left, were assessed together. Every item was scored on a scale of 0 to 2, with a higher score indicating better balance. Total performance, based on the combined score of all items (ranging 0–28), was calculated for each participant [7].

The 16-item Falls Efficacy Scale International (FES-I) is a self-report questionnaire about the fear of falling, which is an issue in older adults because it affects activities of daily living. All FES-I items, ranging from 1 (not in the least concerned) to 4 (very concerned), were assessed at baseline and for 3–12 months [20]. All functions, scored as motor and cognitive, were further sub-scored under different categories (e.g., self-care, sphincter control, transverse, locomotion, social and communication, and walking up or down edgeways) [20]. The combined score, ranging from 7 (no concern about falling) to 28 (severe concern about falling), was calculated [20].

The BBS contains 14 items that assess dynamic and static balance. Each item was scored between 0 (impossible) and 4 (normal function). In this test, the participants were asked to conduct the following actions: (1) sitting to standing; (2) standing unsupported; (3) sitting with back unsupported and feet on the floor or on a stool (instructions: “Please sit with your arms folded for 2 min”); (4) standing to sitting; (5) transfers; (6) standing unsupported with the eyes closed; (8) reaching forward with an outstretched arm while standing; (9) picking up objects on the floor in the standing position; (10) turning the face to look behind, over the left and right shoulders while standing; (11) turning 360°; (12) alternating the foot on a stool or step while standing unsupported; (13) standing with one foot unsupported in the front; and (14) standing on one leg. The maximum possible total score was 56 points. The interpretation of the results was as follows: ≤20 points (wheelchair user), >20 and ≤40 points (walking with assistance such as walking aids), and >40 and ≤56 (independent). A score of ≤46 points indicated that the participant was at a high risk for falls [21]. 

### 2.4. Variables and Comorbidities

Participants’ demographic data, including age, sex, BMI, level of education, occupation, smoking habit, and medical information, were collected for the study. Comorbidities included hypertension, heart disease, diabetes mellitus type 1 and 2, back pain, cancer/tumor, dyslipidemia (disturbance in blood cholesterol levels), lung disease, chronic obstructive pulmonary disease, chronic kidney disease or renal failure, anemia or other blood disease, osteoporosis, history of fracture, neurological disorders, and history of falls.

### 2.5. Psychometric Measurements

To assess the test–retest and inter-rater reliability, 30 participants were assessed at home by a qualified physiotherapist using the Mini-BESTest with a 10-day follow-up. The internal consistency and validity assessment were performed on the total sample. The Mini-BESTest scores were measured at the same time as the BBS scores to ensure that each participant was examined equally. The scores were only used once. Each session lasted for approximately 25 min. 

For construct validity assessment, the BBS was used to assess the convergent validity, and FES-I was used to assess divergent validity.

### 2.6. Statistical Analysis

Data were analyzed using Stata version 15.1 (Stata Corp, College Station, TX, USA). Intraclass correlation coefficients (ICC, model 3.1, two-way mixed-effects model) and 95% confidence intervals (95% CI) were computed to assess the test–retest and inter-rater reliability. An ICC > 0.75 was considered appropriate and > 0.9 was considered excellent [22]. The internal consistency of the Arabic version of the Mini-BESTest was evaluated using Cronbach’s alpha. In addition, construct validity (convergent and divergent) of the Arabic version of the Mini-BESTest against the BBS and FES-I test was assessed using Spearman’s correlation coefficients. The correlation coefficient (r) was interpreted as follows: 0–0.25 = little or no relationship; 0.25–0.50 = fair relationship; 0.50–0.75 = moderate to good relationship, and >0.75 = good to excellent relationship [22]. The standard error of measurement (SEM) was calculated as follows: SEM = SD √(1 − ICC)). The minimal detectable change was calculated using the following formula: MDC = SEM ×1.96 × √2. The significance level was set at alpha = 0.05 for all analyses.

## 3. Result

### 3.1. Patient Demographics and Clinical Characteristics

A total of 140 participants were enrolled in this study. Table 1 shows the basic demographic and clinical characteristics of the participants. The average age was 66 years old (range 60–85 years). Fifty-two percent (74/140) of the participants were male. The average score of the Arabic Mini-BESTest and BBS was 22.7 ± 4.7 and 50.2 ± 8.3, respectively (Table 1). A ceiling effect of the Arabic Mini-BESTest was observed with 24 subjects (17%) reaching the maximum score of 28. 

### 3.2. Reliability

#### 3.2.1. Internal Consistency

Cronbach’s alpha was used to assess the internal consistency of the Arabic version of the Mini-BESTest. The results showed that Cronbach’s alpha was 0.930 for all items, which indicated excellent internal reliability of the scale.

#### 3.2.2. Test–Retest and Inter-Rater Reliability 

The Arabic version of the Mini-BESTest had excellent test–retest reliability (ICC = 0.99, 95% CI, 0.98–0.99), and SEM = 0.45, MDC = 1.23, and inter-rater reliability (ICC = 0.93, 95% CI, 0.70–0.97), and SEM = 0.95, MDC = 2.65.

### 3.3. Validity 

**Figure 2:**
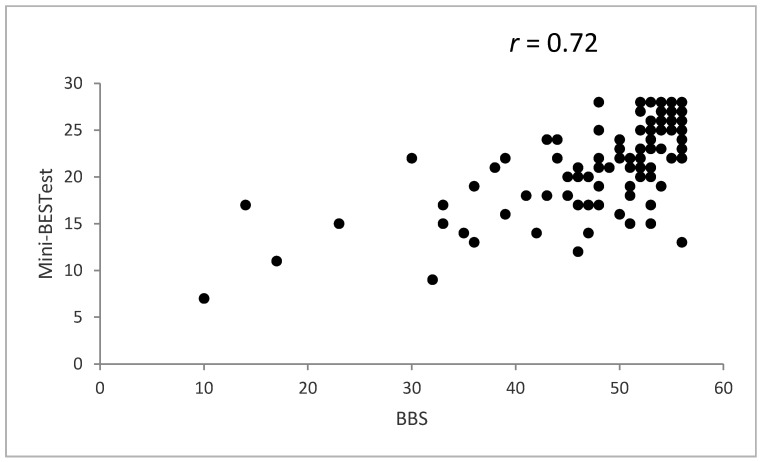
Spearman’s correlations between the Arabic version of the Mini Balance Evaluation Systems Test (Mini-BESTest) and the Berg Balance Scale (BBS).

**Figure 3:**
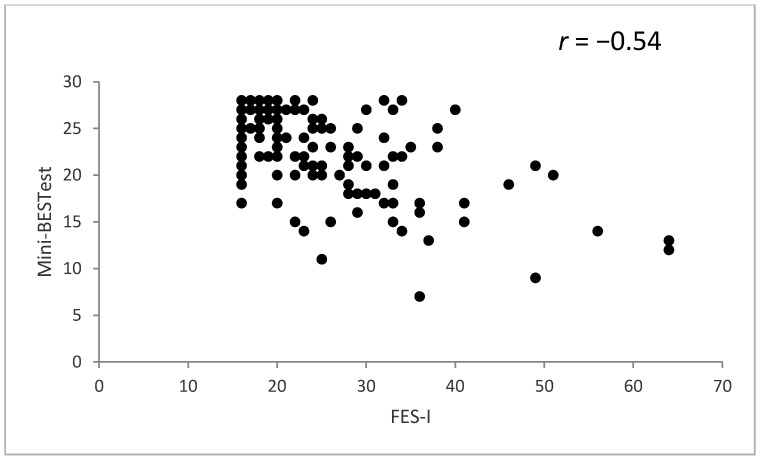
Spearman’s correlations between the Arabic version of the Mini Balance Evaluation Systems Test (Mini-BESTest) and Falls Efficacy Scale International (FES-I).

The Arabic version of the Mini-BESTest had a significant positive association (convergent validity) with the BBS (*r* = 0.72, *p* < 0.01) and a significant negative association (divergent validity) with the FES-I (*r* = −0.54, *p* < 0.01) (Figure 2 and Figure 3).

## 4. Discussion

To the best of our knowledge, the present study was the first to examine the cross-cultural translation and validation of the Mini-BESTest among older community-dwellers living in Saudi Arabia. Our findings showed that the Arabic version of the Mini-BESTest was valid and reliable. Test–retest reliability, measured by the ICC, and internal consistency, measured by Cronbach’s alpha, were excellent. Our results also showed a significant convergent and divergent validity, that was indicated in the significant correlation between our version of the Mini-BESTest and the BBS and FES-I, consistent with previous reports [12,13,16]. In most cases, a higher FES-I score indicated a higher risk of fall. However, the ceiling and floor effects of the Arabic version of the Mini-BESTest were high. 

A previous study revealed that the Greek version of the Mini-BESTest is valid and highly reliable in both inter-rater and test–retest measurements [12]. This was similar to the Arabic version and may be because the total scores and item scores of both studies showed correlations. In addition, the Greek version of the Mini-BESTest is further validated by the strong correlation found between each item in the test, as well as its correlation with the TUG, Functional Reach Test, and FES-I. These findings suggest that the Greek Mini-BESTest warrants further validation studies for other neurological conditions, including multiple sclerosis and PD. In the Greek study, no ceiling or floor effects were observed as the participants had various balance disorders. This contrasted with our study, where the ceiling effect was high because the participants were healthy older adults. 

The validity and reliability of the Spanish version of the BESTest and Mini-BESTest were examined in healthy older community-dwellers [13]. In this study, the psychometric properties were examined in relation to the BBS and FES-I also. Their analysis revealed that the Spanish versions are reliable and valid measures of balance performance. 

Our results were also comparable to those obtained in a study of healthy older adults with the French version of the Mini-BESTest [16]. However, while our Arabic version of the Mini-BESTest had high test–retest reliability, Cronbach’s alpha was used to estimate the internal consistency of each evaluation item in the French study and indicated only an acceptable reliability, but better inter-rater reliability (ICC = 0.974, 95% CI = 0.934–0.99). Additionally, while significant associations with the BBS and FES-I were documented in our study, the approach used in the French study ensured that face and content validity were preserved, allowing for the comparison of the original with translated versions.

A cross-cultural adaptation and validation study of the Mini-BESTest was also conducted in Germany in individuals after stroke [14]. This study showed that the German version has excellent construct validity, high internal consistency, and significant correlations with the BBS and TUG. This high level of internal consistency in the German version was similar to that observed in our study. However, no significant floor or ceiling effects were observed in the German study, which might be explained by differences in the patient cohorts [14].

The validity of the Swedish version of the Mini-BESTest was also investigated in patients with mild or moderate PD or chronic stroke [15]. A high correlation between the Mini-BESTest and BBS results was also observed in this study. In fact, both Swedish and Arabic versions of the Mini-BESTest showed high correlations with the BBS and FES-I. These findings not only suggest that the Mini-BESTest in Swedish has high validity but also that additional validation studies for PD or chronic stroke are warranted. Additionally, a ceiling effect in the BBS scores was observed in the PD group: two participants (22%) reached the maximum score of 56 and four other participants (44%) scored above 50 points. The Mini-BESTest scores also showed a high correlation with TUG scores and a lower correlation with the FES-I scores in the PD group. However, the Swedish version illustrated no overall ceiling effect, as opposed to the high ceiling effect observed in our study in healthy older adults. This study also assessed various balance control systems, further demonstrating that the Mini-BESTest is a well-validated balance assessment tool for the neurological rehabilitation of patients with PD and chronic stroke.

No floor or ceiling effects were observed in the Persian version of the Mini-BESTest administered to PD patients [17]. This observation is consistent with those of the Swedish and English versions. The construct validity in both the Persian and Arabic versions of the Mini-BESTest was assessed against the BBS, a well-accepted balance measurement scale. The Persian version also showed strong correlations with BBS. Furthermore, the average Mini-BESTest scores of non-fallers were significantly higher than those of fallers. This demonstrated that the Mini-BESTest could distinguish PD fallers from non-fallers. 

Compared to the Arabic version of the Mini-BESTest, the Italian version has low reliability indices [7]. The validity of the dynamic Mini-BESTest in Italian is high because many questions belong to a well-known battery of balancing tests. There were no duplicate items or significant ceiling effects in the Italian study.

Similar to our findings, a Turkish study of 84 patients with chronic stroke found a moderate positive correlation between the Mini-BESTest and the BBS [18]. Both Arabic and Turkish versions of the Mini-BESTest had similar reliability, validity, and internal consistency, which could be a result of the appropriate use of standard measurements and correlation analyses to evaluate the validity of the scale. In fact, the test–retest reliability of these two versions had similar correlation coefficients. All items were validated with high reliability using the Cronbach’s alpha coefficient. While the Turkish study focused on patients with chronic stroke, our study focused on healthy older adults. 

### 4.1. Clinical Implications

The Arabic version of the Mini-BESTest may facilitate balance assessments in older adults and help clinicians and researchers use this scale in future research. The Mini-BESTest shows moderate responsiveness for detecting treatment-related improvements in dynamic balance. Its use was recently suggested by an expert panel as part of a balance assessment requirement in adults [7]. However, inadequate knowledge about the quality of the Mini-BESTest across different patient populations is a major obstacle in adopting it into regular clinical practice. In this regard, clinicians have emphasized that the minimum clinically meaningful differences, which indicate progress for patients and care providers, can play an important role in facilitating the adoption of outcome measures [23]. To date, the use of the Mini-BESTest in balance research has been confined to reports of its reliability, validity, and capacity to distinguish faller status [24]. There is a need to validate the results of the Mini-BESTest so that patients and physicians can further understand its clinical importance.

### 4.2. Study Strengths, Limitations, and Future Research Directions

To the best of our knowledge, this study is the first to employ the Arabic version of the Mini-BESTest. Furthermore, our study participants were recruited from different areas in Saudi Arabia. As such, the good validity and reliability of the Arabic version is applicable to the general population, with a focus on the evaluation of psychometric properties of the test in healthy older adults. In this regard, correlations between the Mini-BESTest, BBS, and FES-I results were documented. 

Our study also has some limitations. First, our sample size was small and based on the design of a cross-cultural translation study, with 100 to 250 participants [25]. Second, we did not evaluate patients with illnesses, including neurological diseases. Third, inter-rater reliability should be interpreted carefully as the CI was wide compared to the test–retest reliability; however, our results were similar to previous findings [13]. To accurately estimate the inter-rater reliability, this would require a larger sample size than the one in the current study. Fourth, the sensitivity and responsiveness of the Arabic version of the Mini-BESTest were not evaluated. In future studies, a fully randomized sampling method and a focus on the evaluation of the responsiveness, predictive validity, and specificity in different patient populations, such as those with multiple sclerosis, PD, and stroke, could be explored. 

## 5. Conclusions

In summary, our study demonstrated that the Arabic version of the Mini-BESTest is an accurate assessment tool for dynamic balance in healthy older adults. The Arabic version of the Mini-BESTest is valid and reliable but exhibits some ceiling and floor effects. Significant inverse correlations between the Mini-BESTest and BBS and FES-I results were documented, in which older age was associated with a lower quality of balance and a higher perceived risk of falling. The addition of this Arabic version of the Mini-BESTest to the library of balance measures may allow researchers and clinicians to select the appropriate assessment tool to measure balance in healthy older adults as well as those with balance impairments. The application of the measure in those with balance impairments will require additional validation studies with a larger sample size of specific patient populations. We strongly recommend the application of the Mini-BESTest in different cross-cultural contexts with older community-dwellers with different life expectancies.

## Figures and Tables

**Table 1:** Participant demographics and clinical characteristics (n = 140).

Variable	Mean ± SD
**Age (y)**	66.2 ± 6.2
**Gender, *n* (%)**	
Men	74 (52.8)
Women	64 (45.7)
**Level of education, *n* (%)**	
No formal education	11 (7.8)
Primary school	49 (10.6)
Middle school	19 (46.8)
Secondary school	21 (42.5)
University	40 (28.5)
**Marital status, *n* (%)**	
Single	2 (1.4)
Married	130 (92.8)
Divorced	2 (1.4)
Widowed	6 (4.2)
**Occupation, n (%)**	
Employed	6 (4.2)
Retired	71(50.7)
Unemployed	63 (44.3)
**Smoking, *n* (%)**	
Yes	24 (17.1)
No	116 (82.8)
**Number of comorbidities, *n* (%)**	
None	22 (15.7)
1	34 (24.2)
2 or more	84 (60)
**Number of falls, *n* (%)**	
None	123 (87.8)
1	8 (5.7)
2 or more	9 (2.1)
**Mini-BESTest, mean score (SD)**	22.79 (4.7)
**BBS, mean score (SD)**	50.2 (8.3)
FES-I*, mean score (SD)	24.8 (9.6)
**BMI (kg/m^2^), mean (SD)**	29.1 (5.1)

Mini-BESTest: Mini Balance Evaluation Systems Test; BBS: Berg Balance Scale; FES-I* Falls Efficacy Scale International; BMI: body mass index; SD: standard deviation.

## Data Availability

The datasets used and/or analyzed during the current study are available from the corresponding author on reasonable request.
